# Supplementary material for: The drivers of mental health service utilisation among public primary healthcare workers in Negeri Sembilan
Source: PLoS One. 2025 Jan 31;20(1):e0317654. doi: 10.1371/journal.pone.0317654 (PMC11785275; doi:10.1371/journal.pone.0317654)
Supplement: Attachment — Submitted filename: 1. Response to reviewers_8.9.24.pdf [file pone.0317654.s001.pdf]

Date: 8 September 2024

Dear Chief Editor,

Response to Reviewers and Resubmission of Corrected Manuscript

Thank you for your insightful comments and the valuable feedback provided by the reviewers regarding our manuscript titled "The drivers of mental health service utilisation among public primary healthcare workers in Negeri Sembilan" We have carefully addressed each comment, and revisions have been made to the manuscript accordingly. Please find below a table summarizing the changes made in response to the reviewers' comments.

Table list of corrections made in response to reviewer's comments

No.	Sections	Comments from reviewer	Corrected page no.
1.	Introduction	it is unclear what the authors mean by target 3.8 in sustainable developmental goals. It would be helpful if the authors could explicitly state what the specific goal or target is that they are referring to in here	Page 3
2.		The introduction is very lengthy. Consider reducing unnecessary information.	Page 3-5
3.		Please consider restructuring this sentence to "The systematic review of mental health among HCWs managing the COVID-19 pandemic revealed varying prevalence rates: anxiety had the highest rate at 67.6%, followed by stress at 62.9% and depression at 55.9%. While the lowest rates were observed for depression at 12.1%, stress at 29.8%, and anxiety at 24.1%".	Page 3
4.		Abbreviated and full form of MHSU should be provided at first mention.	Page 4
5.		The paragraph discussing the impact of low MHSU contains a lot of repetitive information about the negative consequences experienced by HCWs.	Paragraphs were condensed. Page 4
6.		Recommend shifting the details about the factors influencing this model to the methods section. By doing so the content will be streamlined, making it easy to understand and avoiding any unnecessary details in the introduction.	Shift to methodology, Page 5&6
7.	Methodology	Please consider briefly elaborating what the Cronbach's alpha and Cohen's kappa values represent.	Page 9,study instruments section

8.		Confidence interval abbreviation can be added in the data analysis part	Page 10, data analysis section
9.		Was an external review board certification/permission required for this study?	Ethical approval obtained from MOH, Malaysia, NMRR-ID-22-02258-CTA (IIR). Page 10&11
10.	Results	Definitions for income categories B40, M40, and T20 need to be provided within the results text.	Page 13
11.	Discussions	The sentence, "The high prevalence... accounted for 96.3%." is ambiguous. Explicitly state whether the 96.3% refers to the percentage of total MHSU or any other measure to ensure clarity	Page 18
12.		This is consistent with the findings of a systematic review and meta-analysis on mental health screening...” is very confusing. It is not clear what specific results or points the authors are referring to.	Page 21
13.		Recommend divide the discussion into further subsections like “impact of mental health training”, “socioeconomic factors”, etc.	Page 18-22
14.	Recommendation	Incorporate a brief paragraph on future directions for investigators and how more rigorous analysis could be used.	Page 23

We hope that the revisions and responses adequately address the concerns raised by the reviewers. We are committed to improving the manuscript and are open to further suggestions. Thank you again for the opportunity to refine our work.

Sincerely,

[Norhafizah Mohd Noor]

1st author
Department of Community Health
Faculty of Medicine and Health Sciences
University Putra Malaysia
